# Favorable response to PD-1 inhibitor plus chemotherapy as first-line treatment for metastatic follicular dendritic cell sarcoma of the spleen: a case report

**DOI:** 10.3389/fimmu.2023.1228653

**Published:** 2023-08-25

**Authors:** Jielang Li, Min Ren, Feng Bi, Ye Chen, Zhiping Li

**Affiliations:** ^1^ Division of Abdominal Tumor Multimodality Treatment, Cancer Center, West China Hospital, Sichuan University, Chengdu, Sichuan, China; ^2^ Department of Radiation Oncology, Cancer Center, West China Hospital, Sichuan University, Chengdu, Sichuan, China; ^3^ Division of Abdominal Cancer, Department of Medical Oncology, Cancer Center and Laboratory of Molecular Targeted Therapy in Oncology, West China Hospital, Sichuan University, Chengdu, Sichuan, China

**Keywords:** PD-1 inhibitor, immunotherapy, follicular dendritic cell sarcoma, sintilimab, chemotherapy

## Abstract

Follicular dendritic cell sarcoma (FDCS) is an uncommon low-grade malignant sarcoma. For localized FDCS, surgery is the most commonly recommended therapy option. However, there is no standard treatment protocol for metastatic FDCS. Here, we present a 68-year-old female with primary spleen FDCS who had multiple peritoneal metastases. She was treated with sintilimab (PD-1 inhibitor) plus chemotherapy (epirubicin plus ifosfamide) as first-line treatment achieving partial response (PR) and a relatively long progression-free survival (PFS) of 17 months. This case suggests that PD-1 inhibitor plus chemotherapy as first-line therapy seem to be a promising treatment option for metastatic FDCS.

## Introduction

Follicular dendritic cell sarcoma (FDCS) is an extremely rare low-grade malignant sarcoma that originates from follicular dendritic cells. Most FDCS arises from lymph nodes, with the cervical, axillary and intra-abdominal lymph nodes being the most frequently affected. Less than one-third of cases occur in extra-nodal sites, such as the liver, lung, tonsil, nasopharynx, pancreas and spleen (1, 2).

Due to the rarity of this disease, no standard treatment protocol exists. For patients with localized disease, most received surgery with or without adjuvant therapy. In clinical practice, chemotherapy is mostly used for patients with metastatic FDCS. Commonly used chemotherapy regimens have included CHOP (cyclophosphamide, vincristine, doxorubicin, prednisolone), ICE (ifosfamide, carboplatin, etoposide), ABVD (doxorubicin, bleomycin, vincristine, dacarbazine) and gemcitabine plus taxane (2–4). However, the efficacy of chemotherapy is limited, and the 2-year survival rate for distant metastatic diseases is approximately 40% (2). Therefore, more effective drugs need to be found.

Programmed death-1 (PD-1)/programmed death factor ligand-1 (PD-L1) checkpoint inhibitors are the main strategies of immunotherapy and have made breakthrough progress in the treatment of various cancers (5–9). However, there are only a few studies on the use of PD-1/PD-L1 inhibitors in patients with FDCS (10–13). Here, we present the first case of metastatic FDCS with high tumor PD-L1 expression and abundant tumor-infiltrating lymphocytes (TILs) in the tumor microenvironment that achieved partial response (PR) and a long progression-free survival (PFS) of 17 months after receiving PD-1 inhibitor plus chemotherapy as first-line treatment.

## Case presentation

**Figure 1:**
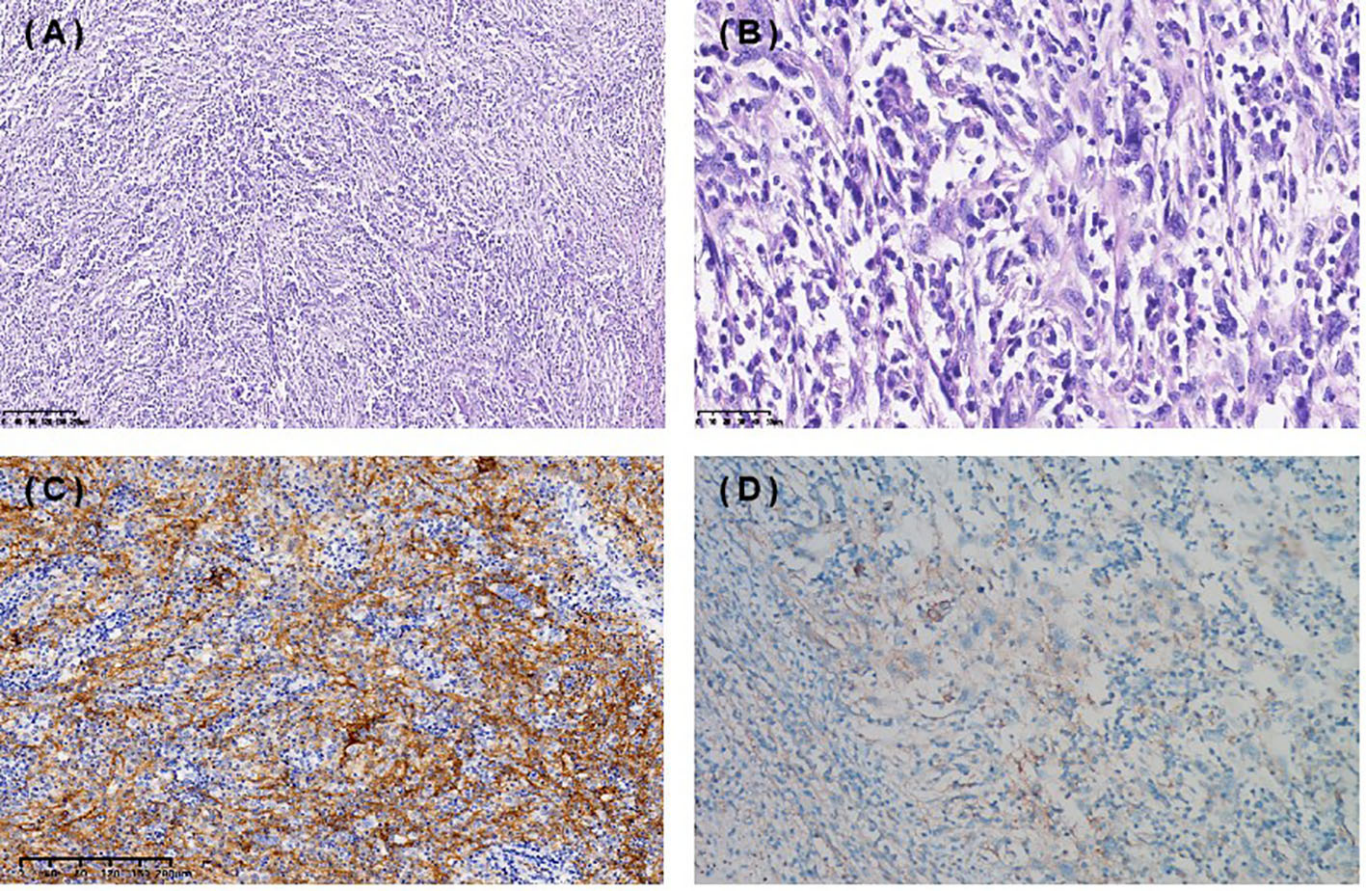
Immunohistochemical staining (IHC) of spleen follicular dendritic cell sarcoma (FDCS). Hematoxylin and eosin staining 10X **(A)**, 40X **(B)** . **(C)** Tumor cells were positive for CD35. **(D)** PD-L1 TPS 1%, CPS 10.

**Figure 2:**
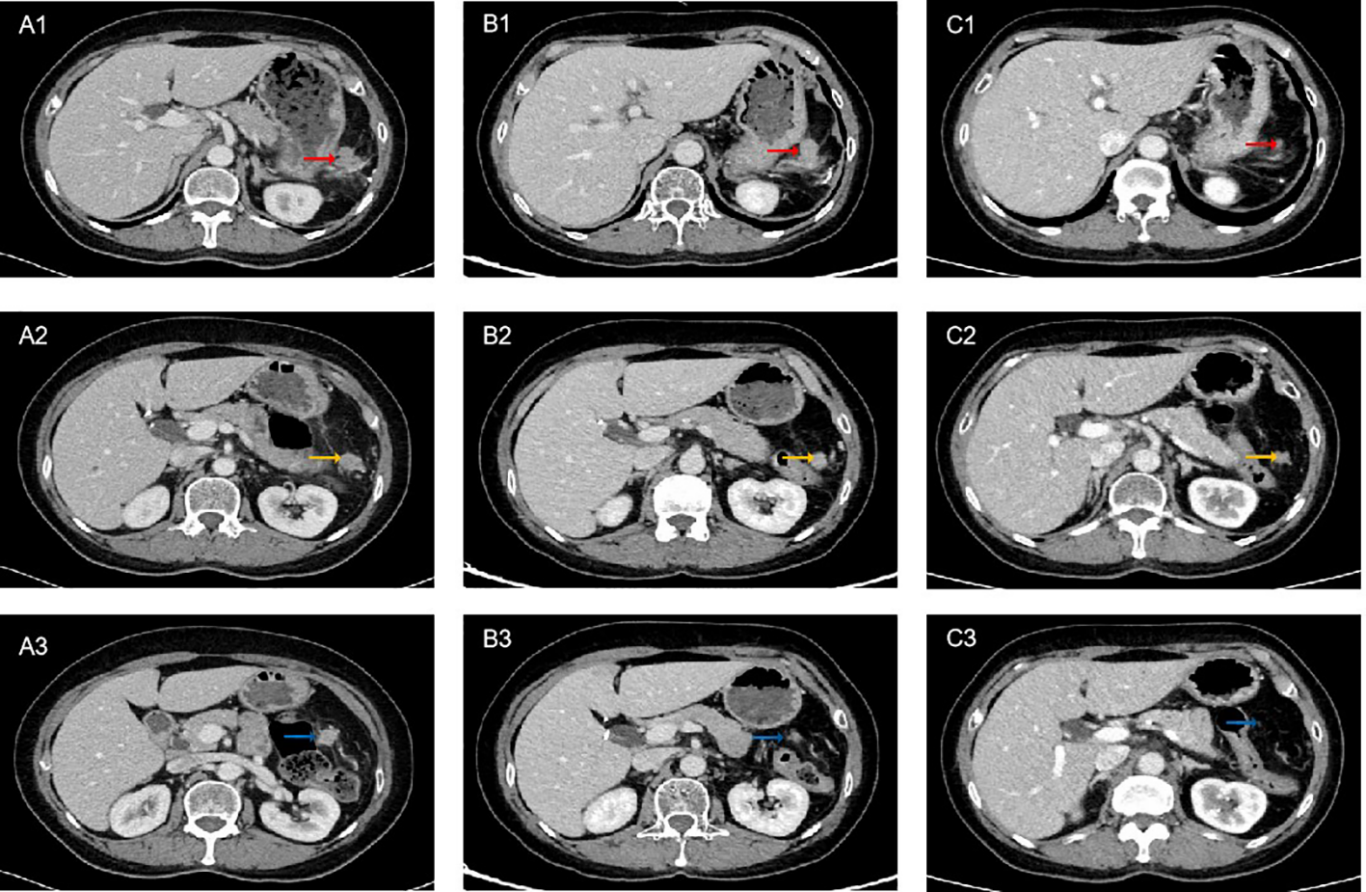
Computed tomography (CT) scan of the patient on treatment. **(A1-A3)** Pretreatment. **(B1-B3)** After 5 cycles of PD-1 inhibitor plus chemotherapy, CT showed partial response (PR). **(C1-C3)** After 8 cycles of PD-1 inhibitor plus chemotherapy combined with radiotherapy, CT showed sustained PR. The red arrows indicate lesion 1, yellow arrows indicate lesion 2, and blue arrows indicate lesion 3.

A 67-year-old female presented with recurrent episodes of fever for 20 days in July 2020. She went to the hospital, and a computed tomography (CT) scan showed a mass lesion with a size of 8.1 cm*7.2 cm in the spleen. On August 28, 2020, she underwent splenectomy. Postoperative pathology showed that the tumor was positive for CD35, CD21, CD23 and CD20 (**Figures 1A-C**). The Ki-67 expression index was 20-30%, and EBER1/2-ISH was positive. Pathological diagnosis confirmed it to be follicular dendritic cell sarcoma of the spleen. She did not receive adjuvant chemotherapy or radiation after surgery. On the follow-up after one year, CT in October 2021 (**Figures 2A1-A3**) indicated extensive intraperitoneal metastases, including in the original splenic zone and beside the left-side colon (the largest nodule size was 3.2*2.6 cm). Surgical debulking of the lesions was not considered feasible by the surgeons.

**Figure 3:**
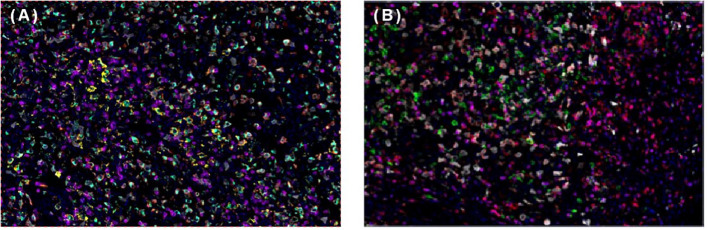
Multiplex immunofluorescence of spleen follicular dentritic cell sarcoma (FDCS). **(A)** Immunofluorescence staining of PD-1 (green), PD-L1 (yellow), CD8 (magenta), CD68 (cyan), and CD163 (red). **(B)** Immunofluorescence staining of CD3 (magenta), CD4 (red), CD20 (green), CD56 (cyan), and FoxP3 (yellow).

To help establish the treatment for her, immunohistochemical (IHC) staining of PD-L1 (**Figure 1D**) and multiple immunofluorescences to evaluate the tumor microenvironment (TME) of this patient in both spleen FDCS cells and tumor stromal cells (**Figure 3**) were carried out. The tumor proportion score (TPS) was 1%, and the combined positive score (CPS) was 10. The data indicated that the tumor cells expressed a high level of PD-L1. Moreover, a relatively high density of infiltrating CD8+ T cells was also observed in tumor cells (2.20%) and stromal cells (3.82%), indicating tumor-infiltrating lymphocyte (TIL) positivity. The analysis revealed that the tumor of this patient expressed both PD-L1 and TILs, indicating the presence of “adaptive immune resistance”. Meanwhile, the low levels of PD-1+CD8+ (0.12% in tumor cells and 0.04% in tumor stromal cells) and CD4+FoxP3+ (0% in both tumor and tumor stromal cells) showed that the inhibitory function of Treg cells was weak. Furthermore, tumor-associated macrophages (TAMs) mainly include two functional states, M1 (anti-tumour) and M2 (tumor-promoting), and for this patient, the proportion of M1-type macrophages (1.59%) was higher than that of M2-type macrophages (0.75%). The above information demonstrated that this patient might be more likely to benefit from immune checkpoint therapies. Next-generation sequencing (NGS) testing results showed low tumor mutation burden (TMB-L) (0.96 Muts/Mb, 8%) and microsatellite stability (MSS).

The patient decided to undergo immunotherapy combined with chemotherapy as palliative first-line treatment. On Oct 12, 2021, she received her first cycle of the AI (epirubicin plus ifosfamide) chemotherapy regimen plus sintilimab (anti-PD-1) every 3 weeks thereafter. After 5 cycles of combined treatment, a CT scan on March 5, 2022 (**Figures 2B1-B3**) showed a decrease in the size of the nodules (the largest nodule size was 2.5*2.4 cm). The efficacy was partial response (PR) based on Response Evaluation Criteria In Solid Tumors (RECIST) v1.1. After eight cycles of PD-1 inhibitor plus chemotherapy, the patient continued to receive maintenance sintilimab monotherapy once every 3 weeks. After achieving PR, the surgeons considered there was still no indication for surgery. To achieve better local tumor control, radiotherapy was administered to her from June 17, 2022, to August 8, 2022 (60 Gy/30 f). Persistent PR was observed after 8 cycles of immunotherapy combined with chemotherapy plus radiotherapy (**Figures 2C1-C3**). Until March 2023, the follow-up CT scan showed disease progression in the hepatic hilar lymph node. The progression-free survival (PFS) was 17 months following sintilimab plus chemotherapy as first-line treatment. During the treatment period, the patient experienced treatment-related adverse events of grade 2 leukopenia and grade 2 hypothyroidism, and her general condition was good.

## Discussion

To the best of our knowledge, this is the first report that metastatic FDCS had a good response and long PFS to a combination of anti-PD-1, chemotherapy, and radiotherapy as first-line treatment.

FDCS was first described by Monda in 1986 (14). Gatta G documented the incidence of FDCS as 0.05/10,0000/year (15). It occurred mainly in adults, and there was no sex difference (2). The etiopathogenesis of FDCS remains unclear. It often manifests as slow-growing, asymptomatic or painful masses. The diagnosis of FDCS is mainly dependent on IHC features. Tumor cells typically express one or more of the following markers: CD21, CD35, CD23, clusterin and CXCL13. CD21, CD35 and CD23 were positive in our patient. Therefore, the definite diagnosis of FDCS was made based on IHC and histologic microscopic findings for this patient.

FDCS is considered to be a low- or intermediate-grade malignancy (16). Local recurrence was observed in 28% of patients, and 27% of cases experienced distant metastasis (2, 3). Unfortunately, our patient developed distant metastases 1 year after surgery. For patients with metastatic disease, the 2-year survival rate was only 42.8%, and the median survival was 9 months (range 0.25-72 months) (2). There is no standard treatment protocol for FDCS even today. Surgical treatment is the most often used therapy for localized FDCS. The role of adjuvant chemotherapy or radiotherapy is debatable (2, 17–19). For patients with unresectable, recurrent and metastatic disease, therapies are diverse. Chemotherapy with or without radiotherapy is the most frequently used treatment. Chemotherapy regimens for aggressive lymphoma are commonly used, such as CHOP, ABVD or ICE, but there is still no consensus.

The therapeutic landscape of tumors has significantly changed over the last years with the rise of immune therapy, especially the immune checkpoint PD-1/PD-L1-based immunotherapy. Xu et al. reported that 50% of FDCS patients were positive for PD-L1 (20). Seven (54%) of 13 assessable FDCS cases showed moderate to strong membranous staining for PD-L1 (21). About 40-60% FDCS cases exhibited neoplastic PD-L1 expression (22). Over 60% of FDCS cases showed conspicuous reactivity for PD-L1 (23). The expression of PD-L1 in each study is shown in **Supplemental Table 1**. The results above revealed FDSC patients as rational candidates for immunotherapy. Moreover, the role of immunotherapy was explored with variable responses in a few FDCS cases. A patient with primary small intestine FDCS received sintilimab (anti-PD-1) plus lenvatinib (antiangiogenic agent) as third-line treatment, achieving a PFS of 7 months (10). A trial of salvage nivolumab (anti-PD-1) was attempted to treat a patient with liver metastases without any success (11). Lee et al. reported two patients with FDCS who received nivolumab (anti-PD-1) and ipilimumab (anti-PD-L1) with evidence of tumor response (12). A man with FDCS received pembrolizumab (a PD-1 inhibitor) monotherapy as second-line treatment and achieved a good response (13). However, due to the rarity of FDCS, there remains insufficient evidence on the effectiveness of emerging treatment modalities. At present, no metastatic spleen FDCS receiving multimodal treatment, including immunotherapy, chemotherapy and radiation as first-line treatment has been reported.

Our patient had high expression levels of PD-L1 and TILs. Studies have shown that high PD-L1 may be a predictive biomarker for the efficacy of PD-1/PD-L1 therapy (5, 24, 25). Patients with higher TIL density predict favorable outcomes (26). Moreover, PD-L1+/TIL+ tumors are most likely to respond to PD-1/PD-L1 blockade therapy (27). Furthermore, TME analysis revealed the weak Treg cells and a high proportion of tumor-associated macrophage M1 type cells. Treg cells suppress effective tumor immunity, being associated with poor prognosis in cancer patients (28) and can be used as a predictor of the clinical efficacy of anti-PD-1 therapies (29). Increasing levels of M1 macrophages indicate a better prognosis (30). Considering the above factors, this patient was treated with sintilimab. The findings in phase III clinical trials have already confirmed the efficacy of immunotherapy combined with standard-of-care chemotherapy to treat tumors (31–34). The underlying mechanisms of these synergetic results include immunogenic tumor cell death, antiangiogenesis, selective depletion of myeloid immunosuppressive cells, and lymphopenia, which decreases regulatory T cells and makes room for proliferation of effector T cells (35, 36). Therefore, she received immunotherapy combined with chemotherapy. In addition, radiation was reported to be used to control local lesions in patients with FDCS (12, 13). Therefore, radiotherapy was also used for her. Through multiple treatment modalities, this patient achieved PR and a long PFS of 17 months.

## Conclusion

To the best of our knowledge, this is the first case of metastatic spleen FDCS with high expression of PD-L1 and TILs receiving PD-1 inhibitor plus chemotherapy as first-line treatment obtained a long PFS. This case suggests that a combination of immunotherapy and chemotherapy as first-line treatment might be a new therapeutic option for metastatic FDCS patients. We also highlight that PD-L1 and TME analyses are important technologies to assist in treatment choice. Further large prospective studies are warranted to confirm the results.

## Data availability statement

The raw data supporting the conclusions of this article will be made available by the authors, without undue reservation.

## Ethics statement

The studies involving humans were approved by the Ethics Committee of West China Hospital, Sichuan University, China. The studies were conducted in accordance with the local legislation and institutional requirements. The participants provided their written informed consent to participate in this study. Written informed consent was obtained from the individual(s) for the publication of any potentially identifiable images or data included in this article.

## Author contributions

JL, MR, FB, YC, and ZL were responsible for the treatment of this patient. JL was responsible for drafting the article. YC was responsible for article revising. All authors commented on previous versions of the manuscript and approved the final manuscript. All authors contributed to the article.
