# Supplementary material for: Favorable response to PD-1 inhibitor plus chemotherapy as first-line treatment for metastatic follicular dendritic cell sarcoma of the spleen: a case report
Source: Front Immunol. 2023 Aug 25;14:1228653. doi: 10.3389/fimmu.2023.1228653 (PMC10485249; doi:10.3389/fimmu.2023.1228653)
Supplement: Supplementary file 1 [file Table_1.docx]

| Supplemental Table 1. Expression of PD-L1 in FDCS | | | | | |
| --- | --- | --- | --- | --- | --- |
| **Reference** | **No. cases** | **Methods for PD-L1** | **Antibodies of PD-L1** | **PD-L1 positive (n, %)** | **Definition of PD-L1 positive** |
| [20] | 20 | IHC | 405.9A11 | 10 (50) | Membranous staining in ≧5% of tumor cells with 2+ or 3+ intensity |
| [21] | 13 | IHC | 28‐8 | 7 (54) | Staining in more than 5% of the neoplastic cells. |
| [22] | 8 | IHC | SP142 | 3 (38) | >5% of the tumor cells were positively stained |
|  |  |  | E1J2J | 3 (38) |  |
|  |  |  | 28‐8 | 5 (63) |  |
| [23] | 22 | IHC | AP30655PU-N | 14 (64) | Combined IHC score for PD-L1* >1 |
| *Staining intensity (1-3) and number of cells (0:0-5%, 1:6-24%, 2:25-74%, 3:75-100%) | | | | | |
